# Serum albumin and FT3/FT4 ratio as additional co-morbidity parameters to predict mortality as a new approach: The Haseki Scoring Index (updated Charlson Comorbidity Index)

**DOI:** 10.1371/journal.pone.0264724

**Published:** 2022-03-14

**Authors:** Betul Cavusoglu Turker, Fatih Turker, Suleyman Ahbab, Emre Hoca, Meryem Tahmaz, Hayriye Esra Ataoğlu

**Affiliations:** 1 Internal Medicine Clinic, Taksim Training and Research Hospital, Istanbul, Turkey; 2 Internal Medicine Clinic, Haseki Training and Research Hospital, University of Health Sciences, Istanbul, Turkey; Istituto Di Ricerche Farmacologiche Mario Negri, ITALY

## Abstract

**Background:**

Charlson Comorbidity Index (CCI) is the common and valid method to predict mortality by classifying comorbidities such as cardiovascular, metabolic, renal, hepatic, pulmonary diseases, and malignancy. Novel risk factors are not included in the Charlson Comorbidity Index, such as thyroid hormone index (FT3/FT4 ratio) and serum albumin levels. In the present study, we aimed to assess whether the thyroid hormone index and albumin are useful clinical parameters in short and long-term mortality.

**Methods:**

In the retrospective cohort study with a 5 year follow up, the data of 1292 patients who were hospitalized between January 1st–June 30th of 2014 were examined. Three months mortality as short term and 5-year mortality as long term were evaluated.

**Results:**

Three months and 5 years mortality rates for 1064 patients were analyzed. We showed that hypoalbuminemia and thyroid hormone index had statistically significant effects on short and long-term mortality. According to ROC analysis it was demonstrated that the scoring system including biochemical parameters such as thyroid hormone index and serum albumin level was more significant for 3-month mortality. In addition, both scoring systems are equal in demonstrating long-term mortality.

**Conclusion:**

Thyroid hormone index and albumin could improve the prognostic performance of the original Charlson Comorbidity Index in short term mortality. The combined score may offer improvements in comorbidity summarization over existing scores.

## 1. Introduction

Chronic diseases are a major health problem in the world, responsible for more than 60% of the causes of death worldwide [1]. Different comorbidity scores have been created as a predictive tool for mortality but only a few have been validated and widely used. Charlson Comorbidity Index (CCI) which was first introduced by Charlson and colleagues in 1987 is the common and valid method to predict mortality by classifying comorbidities. CCI includes many health problems such as cardiovascular, metabolic, renal, hepatic, pulmonary diseases and malignancy. Each mortality was assigned a score based on the relative risk. Present, CCI is thought to be insufficient in predicting mortality due to advances in the management of chronic diseases and improvements in diagnostic methods [2]. It seems that considering of some clinical conditions and laboratory parameters are important in assessing the survival.

The thyroid hormone index (FT3/FT4 ratio) and albumin are both useful prognostic markers for assessing the mortality of patients. Some recent literature has shown that thyroid functions and serum albumin levels have prognostic value [3–5]. The FT3/FT4 ratio, a thyroid hormone index, could show deiodinase activity, which is responsible for the conversion of T4 to T3. In chronic or acute illness, this conversion declines and it calls euthyroid sick syndrome. Previous studies have demonstrated that thyroid hormone index was strongly associated with increased all-cause of mortality [4]. Van vliet et al. showed that there is a strong relationship between thyroid status and survival in old age. Their study has shown that higher FT3/FT4 ratios, higher FT3 levels, and lower FT4 levels are associated with a lower mortality rate [6]. Therefore, euthyroid sick syndrome has become more important for patients. This syndrome can be not only as a disease but also as a parameter to assess mortality.

Albumin can be used as a nutritional marker in critically ill patients [7]. Previous studies showed a close relationship between the nutritional status and the degree of inflammation; and this association may lead to the devolepment of some complications. Touma and Bisharat determined that serum albumin is strongly associated with long-term mortality in readmitted medical patients and persistant hypoalbuminemia during recurrent admissions is associated with increased risk of long term mortality [8]. Hypoalbuminemia can be a risk factor for higher all-cause mortality.

Previous studies show that low T3 / T4 ratio and hypoalbuminemia are risk factors for all-cause mortality and these novel risk factors are not included in the Charlson Comorbidity Index. In the present study, we aimed to assess whether the thyroid hormone index and albumin are useful clinical parameters in short and long term mortality. In addition, we aimed to compare the prognostic performance of thyroid hormone index and serum albümin levels with using the CCI score as reference standards.

## 2. Methods

### 2.1. Study design and patients

This study which conducted in the internal medicine clinic of Haseki Health Training and Research Hospital was designed to evaluate the medical data records of hospitalized patients between 1^st^ January– 30^th^ June 2014. The study protocol was reviewed and approved by the Institutional Review Board at University of Health Sciences, Haseki Health Training and Research Hospital, (Ref No:23 R /2018). This retrospective cohort study was conducted in accordance with principles of good clinical practice and the declaration of Helsinki. We planned the retrospective cohort study which included 1292 patients who were hospitalized between January 1st–June 30th of 2014 in our inpatient clinic. Patients were followed up for five years about primary endpoint as all cause mortality according to the hospital database. 104 repeated hospitalizations and 24 participants with missing data were excluded. The informed consent was obtained from all patients who were hospitalized at our clinic. The final study population included 1164 (571 male and 593 female) patients with a median available follow-up period of 43 months (min:1-max:65). Data for the study analysis was taken from the electronic hospital management system of Haseki Training and Research Hospital which include demographic information, medical history, patient’s comorbidities, vital signs at admission and diagnostic blood tests. Medical conditions including history of myocardial infarction, congestive heart failure, peripheral vascular disease, cerebrovascular disease, peptic ulcer, chronic liver disease, dementia, chronic obstructive pulmonary disease, connective tissue disorders, diabetes mellitus (complicated or not), kidney disease, malignancies (solid and hematologic) with or without metastases and acquired immunodeficiency syndrome were recorded. Each comorbidity is assigned a score ranging from 1 to 6 and the sum of these scores makes the final CCI score. The CCI score was calculated for each patient by identifying all comorbidities. Follow-up information was collected through data processing system of Haseki Training and Research Hospital. The Turkish National Death Registry was used to confirm mortality reports of the follow-up clinic. Three months mortality was evaluated as short-term mortality and 5-year mortality as long-term mortality. The dependent outcome variable was death during the follow-up year.

Charlson comorbidity index includes chronic ischemic heart disease, congestive heart failure, peripheral vascular disease, cerebrovascular disease, peptic ulcer, chronic liver disease, dementia, chronic obstructive pulmonary disease, connective tissue disorders, diabetes mellitus (with and without complication), kidney disease, malignancies (solid and hematologic) and acquired immunodeficiency syndrome. Haseki Scoring index (updating Charlson Comorbidty Index, u-CCI) includes albumin and FT3/FT4 ratio in addition to CCI. The relative risk of each parameter included in the study affecting short- and long-term mortality was calculated statistically. A score based on relative risk was added to the score of diseases that statistically significantly increased the risk of mortality (S1 Table). Only one point was given to the parameters included in the charlson comorbidity index that did not affect mortality.

### 2.2. Laboratory measurements

Routine blood samples for all laboratory tests were taken after a 12-hour fasting period between 6:00 am and 7:00 am. Blood samples were taken from the patients on the first day of the hospitalization, before the drug treatment started and they analyzed immediately. Plasma biomarkers including liver and kidney functions, blood glucose, serum lipid, hemogram, thyroid hormones, albümin and inflamatory markers including sedimantation and CRP were analyzed laboratory of Haseki Training and Research Hospital. The laboratory findings were obtained from the patients’ electronic medical records. Biochemical parameters were performed for all participants. Albumin level below 2.5, which is the lower limit in our hospital laboratory, was defined as hypoalbuminemia. When the FT3/FT4 ratio (thyroid hormone index) was evaluated according to our median level, it was evaluated as 2.27 and below as low. CCI score was calculated for each patient by identifying all comorbidities.

### 2.3. Follow-up and endpoints

The follow-up period ended in June 2019. All data were verified with the hospital records. Follow-up information was collected through data processing system of Haseki Training and Research Hospital. The primary endpoints of this study were composite outcome, including all-cause mortality, as documented in the database. The main outcome measure (dependent variable) in this study was all-cause-mortality. The Turkey national death registry was used to confirm mortality reports of the follow-up clinic. During the follow-up, a total of 661 participants died. 3-month mortality was evaluated as short-term mortality and 5-year mortality as long term mortality.

### 2.4. Statistical analysis

The primary endpoint of the study was all-cause hospital mortality. Categorical variables are reported as frequencies and number (percentage), and the *X*^2^ test was used to compare groups. Continuous variables are reported as mean SD, and Student t test was used to compare groups. Continuous data were described using the mean and standard deviation for normally distributed data. The distribution of baseline characteristics across categories of the exposure variable was evaluated using parametric and nonparametric statistics as appropriate. The Cox proportional-hazards regression model was used to analyze the effects of the variables on event-free survival. The predictive performance of hypoalbuminemia, the FT3/FT4 ratio, and the CCI score was assessed by Cox regression. As continuous variables, the predictive performance of the CCI score, the CCI score + the FT3/FT4 ratio + hypoalbuminemia was assessed by indices of discrimination. Each model was entered into a logistic regression model to obtain the individual risk probability of all-cause death. Univariate analysis was performed using continuous variables as predictors of mortality, and the area under the receiver operating characteristic curve (AUROC) values were determined from this analysis. Haseki scoring (u-CCI) and Charlson comorbidity scoring values in predicting short and long-term mortality were analyzed using ROC (receiver-operator characteristic) curve analysis. The predictive ability of the models was assessed by calculating the area under the receiver operating characteristics (ROC) curves, along with their 95% confidence intervals, for the CCI score and the Haseki score (u-CCI).

All data were analyzed using Statistical Package for Social Sciences (SPSS) version 17.0 software (SPSS Inc., Chicago, IL, United States), with significance of any p < 0.05. Continuous data were described using the mean and standard deviation for normally distributed data. The distribution of baseline characteristics across categories of the exposure variable was evaluated using parametric and nonparametric statistics as appropriate. The proportions were compared with chi-square test or Fisher’s exact test.

## 3. Results

A total of 1064 patients (593 females and 571 males) who were hospitalized between January 1st–June 30th of 2014 were included in this study. 3- month and 5-year mortality rates were analyzed. The mean age of the study subjects was 65.61±17.6. Mild liver damage was detected in 136 (11.7%) patients and severe liver damage in 107 (9.2%) patients. 79 (6.8%) patients had solid tumors and 69 (5.7%) patients had metastatic solid tumor. Two (0.2%) patients had known HIV infection. Chronic renal failure was detected in 415 (35.7%) patients, congestive heart failure in 279 (24%) patients, chronic obstructive pulmonary disease in 139 (11.9%) patients, peripheral artery disease in 41 (3.5%) patients. 108 (9.3%) patients had cerebrovascular event. 161 patients were diagnosed with diabetes mellitus without end-organ damage, and 297 patients had diabetes mellitus with end-organ damage. Laboratory tests of patients were examined. The median of Charlson comorbidity index was 5 (Tables 1 and 2).

**Table 1:** Baseline characteristics of patients in 3 month mortality.

	Survivor	Mortality in 3 month	P value
Gender F/M	473/431	120/140	0.080
Age	63.30 ± 17.99	73.62 ± 13.07	**<0.001**
Diabetes Mellitus Without end-organ Damage	142 (%15.7)	19 (% 7.3)	**0.002**
Diabetes Mellitus With end-organ Damage	230 (%25.4)	67 (%25.7)	**0.002**
Mild Liver Damage	92 (%10.2)	44 (%16.9)	0.003
Severe Liver Damage	78 (%8.6)	29 (%11.2)	0.214
Solid Tumor Without Metastasis	51 (%5.6)	28 (%10.8)	**0.004**
Solid Tumor With Metastasis	24 (%2.7)	42 (%16.2)	**<0.001**
AIDS	0	2 (%0.8)	**0.008**
Chronic Renal Failure	284 (%31.4)	131 (%50.4)	**<0.001**
Congestive Heart Failure	203 (%22.5)	76 (%29.2)	**0.024**
Ischemic Heart Disease	224 (%24.8)	78 (%30)	0.091
Chronic Obstructive Pulmonary Disease	105 (%11.6)	34 (%13.1)	0.522
Peripheral Artery Disease	30 (%3.3)	11 (%4.2)	0.482
Cerebrovascular Disease	73 (%8.1)	35 (%13.5)	**0.008**
Dementia	30 (%3.3)	35 (%13.5)	**<0.001**
Hemiplegia	12 (%1.3)	11 (%4.2)	**0.003**
Connective Tissue Disease	38 (%4.2)	5 (%1.9)	0.086
Leukemia	7 (%0.8)	6 (%2.3)	**0.038**
Lymphoma	14 (%1.5)	5 (%1.9)	0.675
Peptic ulcer	30 (%3.3)	7 (%2.7)	0.612
Albumin (g/L)	3.41 ± 0.60	2.83 ± 0.57	**<0.001**
The Thyroid Hormone index (Ft3/Ft4 ≤2,27)	2.51 ± 0.87	1.96 ± 0.74	**<0.001**

**Table 2:** Baseline characteristics of patients in 5 year mortality.

	Survivor	Mortality in 5 year	P value
Gender F/M	285/253	308/318	0,301
Age	56.4 ± 18.1	72.92 ±13.2	**<0.001**
Diabetes Mellitus Without end-organ Damage	98 (% 19.5)	63 (% 9.5)	**<0.001**
Diabetes Mellitus With end-organ Damage	97 (% 19.3)	200 (% 30.3)	**<0.001**
Mild Liver Damage	60 (% 11.2)	76 (%12.1)	0.601
Severe Liver Damage	43 (% 8.5)	64 (% 9.7)	0.507
Solid Tumor Without Metastasis	16 (% 3.2)	63 (%9.5)	**<0.001**
Solid Tumor With Metastasis	1 (%0.2)	65 (%10.4)	**<0.001**
AIDS	0	2 (%0.3)	0.189
Chronic Renal Failure	105 (%20.9)	310 (%46.9)	**<0.001**
Congestive Heart Failure	72 (%14.3)	217 (%31.3)	**<0.001**
Ischemic Heart Disease	111 (%22.1)	191 (%28.9)	**0.008**
Chronic Obstructive Pulmonary Disease	38 (%7.6)	101 (%15.3)	**<0.001**
Peripheral Artery Disease	9 (%1.8)	32 (%4.8)	**0.005**
Cerebrovascular Disease	27 (%5.4)	81 (%12.3)	**<0.001**
Dementia	6 (%1.2)	59 (%8.9)	**<0.001**
Hemiplegia	4 (%0.7)	19 (%3)	**0.005**
Connective Tissue Disease	29 (%5.8)	14 (%2.1)	**<0.001**
Leukemia	3 (%0.6)	10 (%1.6)	0.092
Lymphoma	6 (%1.1)	13 (%2.1)	0.197
Peptic ulcer	17 (%3.4)	20 (%3)	0.733
Albumin (g/L)	3.53 ± 0.58	3.08 ± 0.61	**<0.001**
The Thyroid Hormone index (Ft3/Ft4 ≤2,27)	2.67 ± 0.91	2.16 ± 0.76	**<0.001**

In 3 months, 120 female and 140 male patients died (Table 1). When the effects of the presence of chronic diseases in CCI, hypoalbuminemia (≤2.5) and low FT3/FT4 ratio (≤2.27) on mortality were examined with 3 months survival with logistic regression model, we observed that chronic kidney failure (p:0.003 OR:1.69), Ischemic heart disease (P:0.039 or:1.51), demantia (p:0.001 OR:2.9), leukemia (p:0.006 OR:5.97), solid tumor without metastasis (p:0.001 OR:2.49), solid tumor with metastasis (p:0.001 OR:12.37), age between 60–69 (p:0.041 OR:2.17), age between 70–79 (p:0.008 OR:2.61), age over 80 (p:0.001 OR:3.96) were effective risk factors in the 3rd month mortality according to the backward stepwise method. According to cox regression, we found that gender was not statistically significant in short-term mortality (p: 0.080). The presence of solid tumor and metastatic tumor was statistically significant in 3-month mortality (p: 0.006, p: <0.001 respectively). HIV infection was significantly affected short-term mortality (p: <0.001). Chronic kidney failure, dementia, leukemia was found to significantly increase short-term mortality (p: 0.035, p: 0.004, p:0.002 respectively). Ischemic heart disease, liver damage, chronic obstructive pulmonary disease, peripheral artery disease, connective tissue disease, lymphoma and peptic ulcer diseases which are in the Charlson Comorbidity Index, were not statistically significant on short-term mortality (p: 0.069, p: 0.101, p: 0.245, p: 0.682, p: 0.548, p: 0.779 respectively) (Table 3).

**Table 3:** Cox regression of comorbidities, albumin and thyroid hormone index in 3-month mortality.

	P value	HR	95,0% CI For HR	
			Lower	Upper
Age	**0.002**			
Age (<50 / 50–59)	0.402	1.33	0.680	2.61
Age(<50 / 60–69)	0.075	1.77	0.943	3.32
Age(<50 / 70–79)	**0.015**	2.12	1.159	3.88
Age(<50 / ≥80)	**0.001**	2.76	1.513	5.01
Diabetes Mellitus	0.339			
Diabetes Mellitus Without End-organ Damage	0.141	0.79	0.578	1.08
Diabetes Mellitus With End-organ Damage	0.775	0.94	0.602	1.46
Liver Damage	0.101	1.39	0.938	2.05
Solıd Tumor	**<0.001**			
Solid Tumor Without Metastasis	**0.006**	1.81	1.182	2.75
Solid Tumor With Metastasis	**<0.001**	4.84	3.329	7.03
AIDS	**<0.001**	14.36	3.366	61.29
Chronic Renal Failure	0.**035**	1.33	1.021	1.73
Congestive Heart Failure	0.185	1.22	0.908	1.64
Ischemic Heart Disease	0.069	1.31	0.979	1.75
Chronic Obstructive Pulmonary Disease	0.245	1.26	0.856	1.84
Peripheral Artery Disease	0.682	1.14	0.610	2.12
Cerebrovascular Disease	0.371	1.21	0.800	1.82
Dementia	**0.004**	1.83	1.216	2.74
Hemiplegia	0.329	1.41	0.706	2.83
Connective Tissue Disease	0.548	0.74	0.271	1.99
Leukemia	**0.002**	3.92	1.686	9.10
Lymphoma	0.492	1.38	0.554	3.42
Peptic ulcer	0.779	0.89	0.407	1.96
Hypoalbuminemia	**<0.001**	2.62	1.802	3.81
The Thyroid Hormone index (Ft3/Ft4 ≤2,27)	**<0.001**	1.84	1.362	2.49

**Fig 1:**
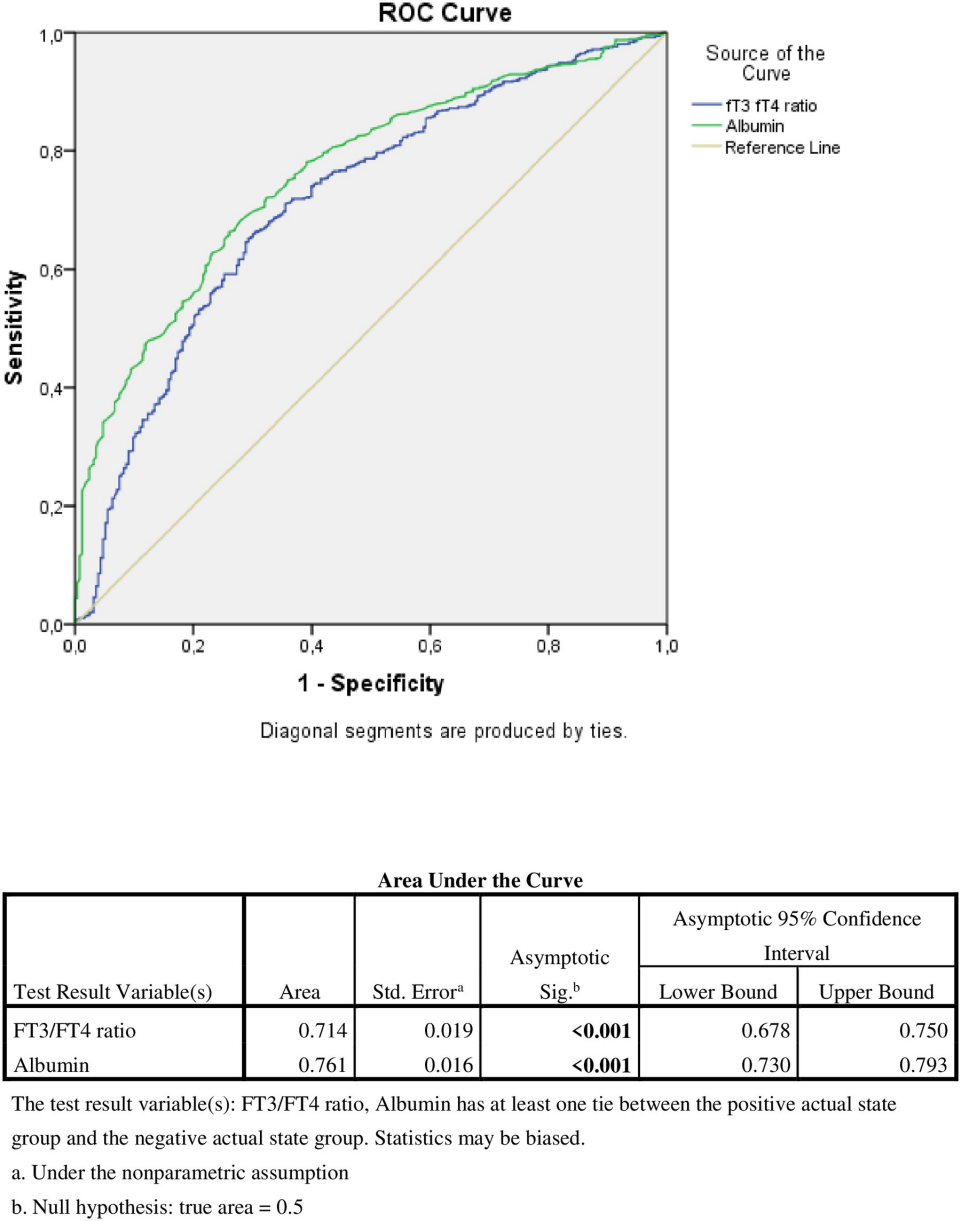
Roc analysis of FT3/FT4 ratio and albumin in 3 month mortality.

It was determined that hypoalbuminemia and thyroid hormone index had statistically significant effects on short-term mortality (p:<0.001) (Fig 1).

**Fig 2:**
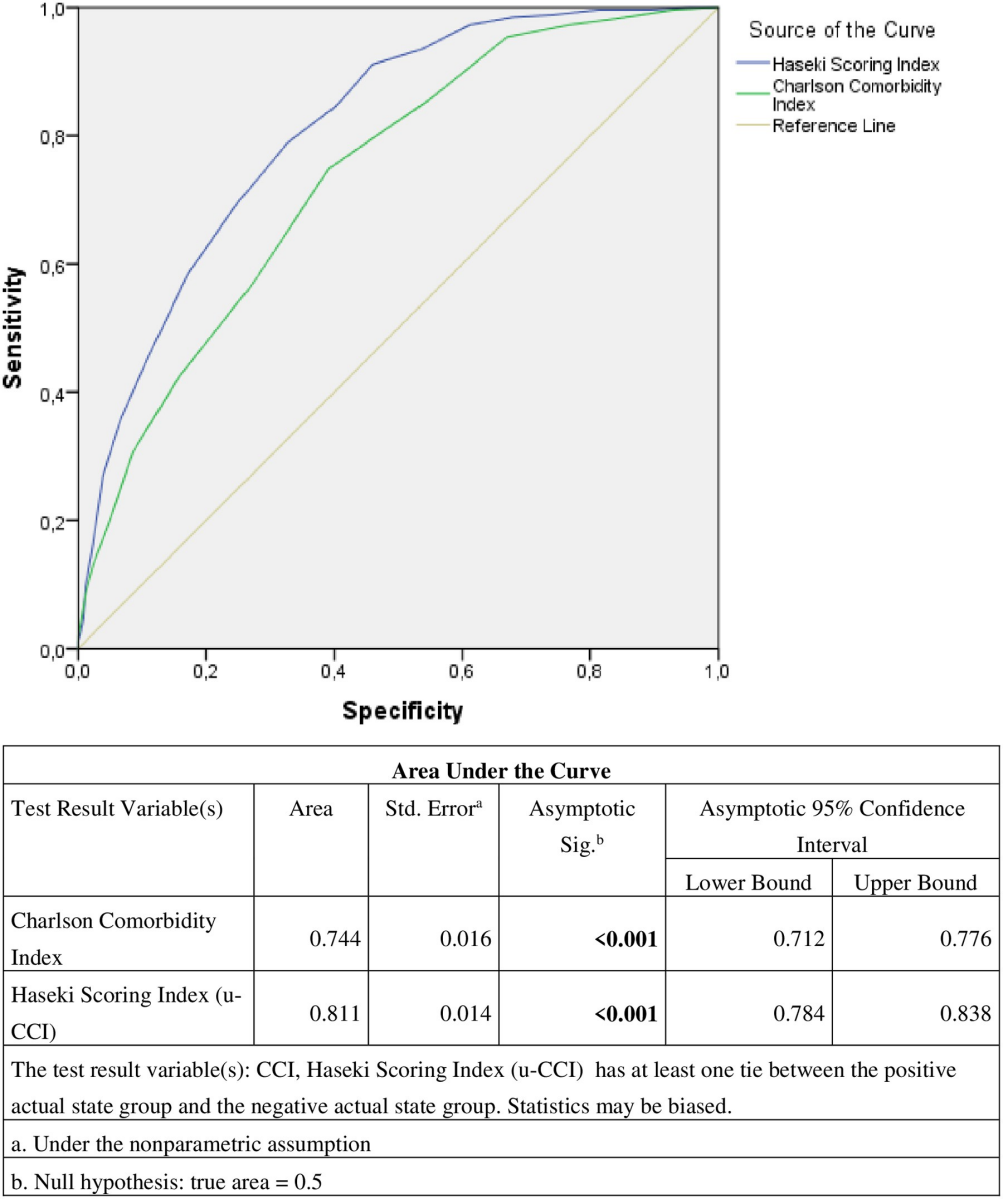
Roc analysis of Haseki Scoring Index (u-CCI) and Charlson Comorbidity Index in 3-month mortality.

Higher Charlson Comorbidity Index score was significantly increasing the 3-month mortality. According to Roc analysis it was seen that the scoring system including biochemical parameters such as thyroid hormone index and albumin was significantly increase 3-month mortality (Fig 2).

328 female and 333 male patients died in the end of the 5th year (Table 2). When the effects of the presence of chronic diseases in CCI, hypoalbuminemia (≤2.5) and low FT3/FT4 ratio (≤2.27) on mortality were examined, in the regression model which was created according to the 5th year survival results it was observed that chronic kidney failure (p:0.001 OR:2.3), congestive heart failure (P:0.01 or:1.89), demantia (p:0.012 OR:3.2), leukemia (p:0.018 OR:6.35), solid tumor without metastasis (p:0.001 OR:4.5), solid tumor with metastasis (p:0.001 OR:128.4), age between 50–59 (p:0.003 OR:2.99), age between 60–69 (p:0.01 OR:2.97), age between 70–79 (p:0.001 OR:4.84), age over 80 (p:0.001 OR:12.26), diabetes with complication (p:0.009 OR:1.67), chronic obstructive pulmonary disease (p:0.003 OR:2.04), peripheral artery disease (p:0.025 OR:2.79) were effective risk factors in the 3rd month mortality according to the backward stepwise method. According to cox regression it was found that gender was not statistically significant in long-term mortality (p:0.199). It was determined that presence of liver damage, lymphoma, connective tissue disease, presence of peptic ulcer, ischemic heart disease and serebrovascularm event did not have a statistically significant effect on 5-year mortality (p:0.092 p:0.061, p:0.160, p:0.754, p:0.201, p:0.138 respectively) (Table 4).

**Table 4:** Cox regression of comorbidities, albumin and thyroid hormone index in 5 year mortality.

	P value	HR	95,0% CI For HR	
			Lower	Upper
Age	**<0.001**			
Age (<50 / 50–59)	0.001	1.98	1.339	2.940
Age(<50 / 60–69)	**<0.001**	2.31	1.583	3.374
Age(<50 / 70–79)	**<0.001**	2.88	2.007	4.131
Age(<50 / ≥80)	**<0.001**	4.32	3.013	6.191
Diabetes Mellitus	0.133			
Diabetes Mellitus Without Endorgan Damage	0.903	1.01	0.839	1.221
Diabetes Mellitus With Endorgan Damage	**0.048**	1.29	1.002	1.665
Liver Damage	0.092	1.25	0.964	1.635
Solid Tumor	**<0.001**			
Solid Tumor Without Metastasis	**<0.001**	1.96	1.483	2.583
Solid Tumor With Metastasis	**<0.001**	6.03	4.531	8.031
AIDS	**<0.001**	20.66	4.999	85.381
Chronic Renal Failure	**<0.001**	1.39	1.171	1.641
Congestive Heart Failure	**<0.001**	1.41	1.173	1.688
İschemic Heart Disease	0.201	1.13	0.939	1.350
Chronic Obstructive Pulmonary Disease	**0.008**	1.36	1.084	1.706
Peripheral Artery Disease	**0.006**	1.68	1.161	2.439
Cerebrovascular Disease	0.138	1.22	0.938	1.592
Dementia	**0.002**	1.61	1.192	2.161
Hemiplegia	**0.026**	1.79	1.074	2.982
Connective Tissue Disease	0.160	0.67	0.386	1.170
Leukemia	**<0.001**	4.03	2.050	7.944
Lymphoma	0.061	1.71	0.976	2.993
Pepticulcer	0.754	0.92	0.564	1.514
Hypoalbuminemia	**<0.001**	1.69	1.408	2.050
The Thyroid Hormone İndex (Ft3/Ft4 ≤2,27)	**<0.001**	1.48	1.243	1.762

**Fig 3:**
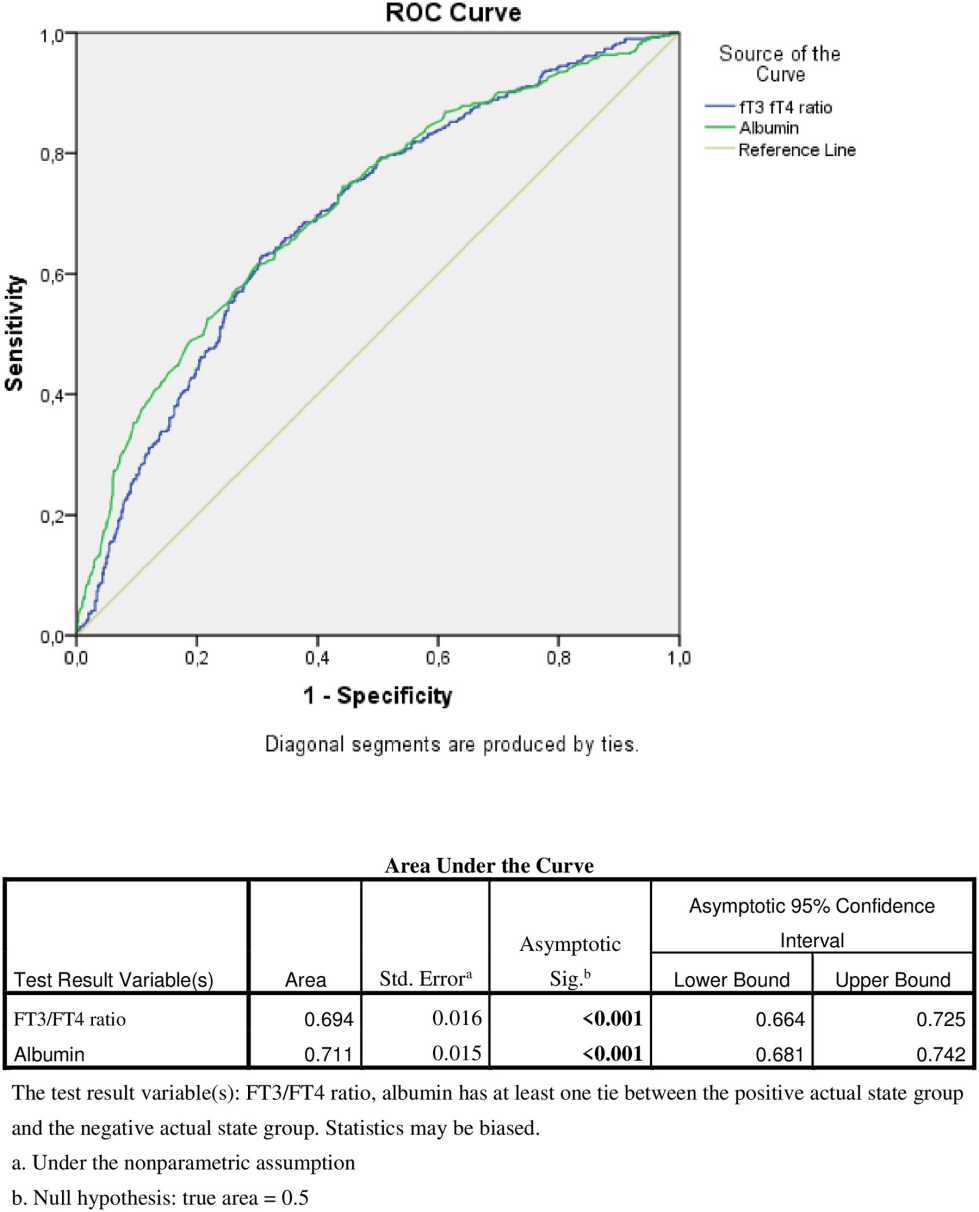
Roc analysis of FT3/FT4 ratio and albumin in 5 year mortality.

Hypoalbuminemia, low FT3/FT4 ratio were statistically significantly increased long-term mortality (p: <0,001) (Fig 3).

**Fig 4:**
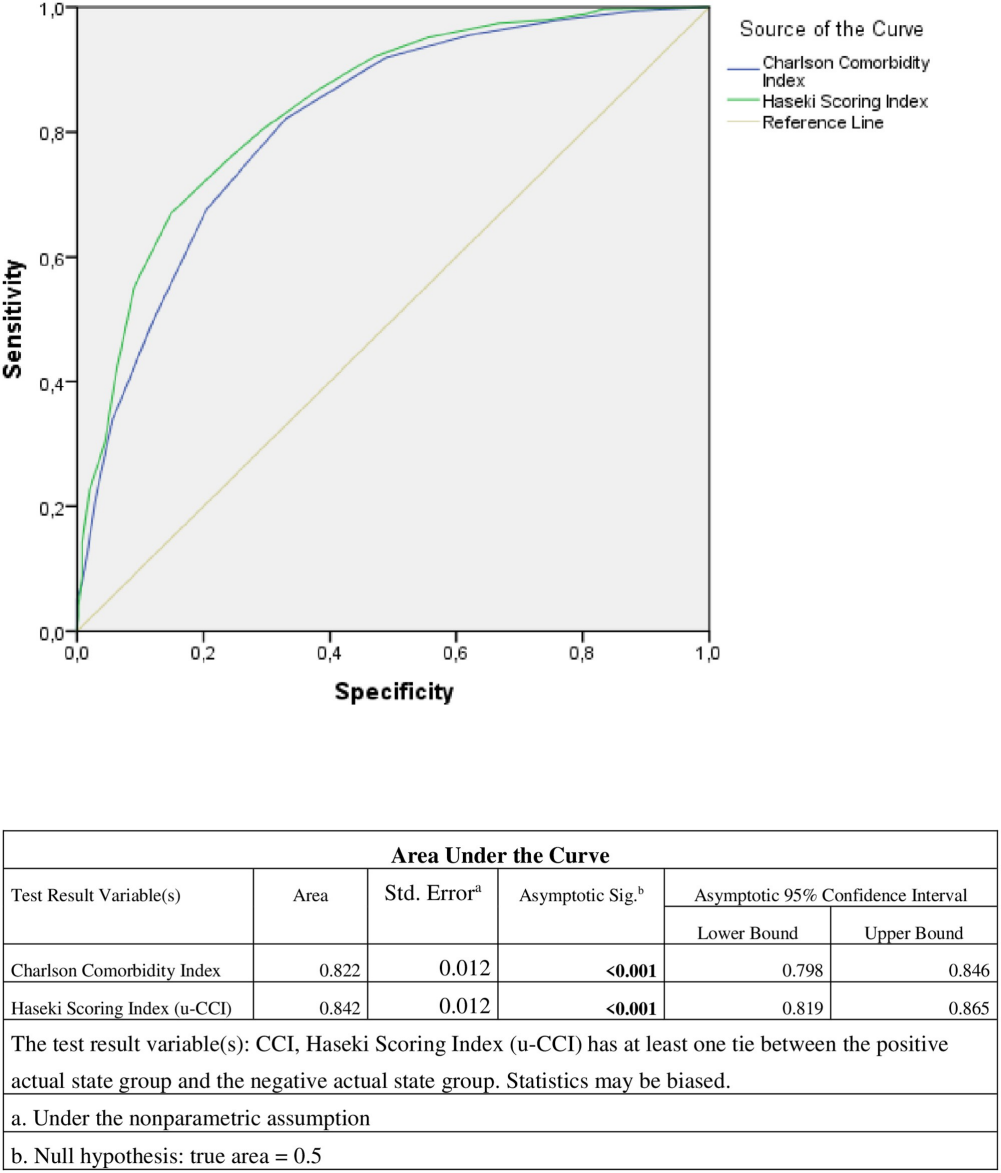
Roc analysis of Haseki Scoring Index (u-CCI) and Charlson Comorbidity Index in 5 year mortality.

Higher CCI Score was found to increase statistically significant long-term mortality (p:0.001). Roc analysis showed that both scoring systems are equal in predicting long-term mortality (Fig 4).

## 4. Interpretation

In our study, 1164 patients who were hospitalized to the internal medicine clinic of our hospital were examined for the comorbid diseases, laboratory tests and their effects on short- and long-term mortality in Charlson Comorbidity Index. Our main findings were as follows: (1) It was found that the 60–69 age, 70–79 age range, 80 and over, diabetes mellitus with organ damage, solid tumor, hemiplegia, metastatic solid tumor, HIV infection, congestive heart failure, chronic obstructive pulmonary disease, chronic kidney failure, dementia, peripheral artery disease, leukemia, low FT3/FT4 ratio, hypoalbuminemia were the most effective risk factors for long term mortality; (2) Solid tumor, chronic kidney failure, AIDS, dementia, leukemia, low FT3/FT4 ratio, hypoalbuminemia were determined as the most effective risk factors for short term mortality; (3) FT3/FT4 ratio and albumin are could improve the prognostic performance of the original Charlson Comorbidity Index in short term mortality.

In the CCI, only comorbid diseases were examined, and it was created according to the presence of comorbid diseases. In our study, unlike charlson comorbidity index, the relationship between albumin levels, FT3/FT4 ratio and mortality were also examined. As it is known, albumin regulates plasma oncotic pressure and capillary membrane permeability and removes free radicals from the body. It is antioxidan molecule for body and a negative acute phase reactant. Serum albumin levels decreases in response to inflammation during acute critical illnesses or chronic systemic diseases [9]. Hypoalbuminemia is the result of the effects of inflammation and malnutrition, in patients with chronic disease [10–12]. Cabrerizo et al. reported that serum albumin level is a good marker of nutritional status in clinically stable individuals [13]. Malnutrition is a worldwide problem in older populations, especially hospitalized patients. Many studies have show that hypoalbuminemia is associated with mortality in patients with various diseases such as acute coronary syndrome [14], chronic kidney disease [15], cancer [14–17] and hip fractures [18]. In previous studies, low albumin levels were associated with an increased risk in all cause mortality [19, 20]. In accordance with our study, it was thought that low albumin increased mortality due to malnutrition and inflammation [19, 20]. For this reason, it was thought that albumin level could be a parameter which is used to assess the short and long-term mortality risk.

It was found that low T3 and high T4 level increased statistically significantly on both short and long-term mortality. T3 and T4 are two main hormones which secreted by the thyroid gland. T3 is thought to be biologically active hormon because affinity of the thyroid hormone receptor is higher for T3 than for T4. Serum T4 is either converted to active T3 which shows receptor mediated effects or converted into inactive rT3 with the enzyme deiodinase in tissues [21–23]. In cases of infectious or inflammatory diseases, this conversion declines, so the level of T3 decreases and the level of rT3 increases [24, 25]. Previous studies have showed that thyroid hormone index could demonstrate deiodinase activity [26]. Pearce et al. showed that low free T3 level increase mortality [4]. Some studies have shown that low free T3 level is associated with chronic diseases in the elderly [26]. The results of Cox regression analysis demonstrated that reduction of thyroid hormone index was associated with 1,480-fold greater of 5 year all-cause death and with 1,843 fold greater of 3 month death.

Presence of peptic ulcer was not found statistically increased in our study as in previous studies [27–29]. It was thought that peptic ulcer would not be a useful and effective comorbidity in predicting mortality.

FT3/FT4 ratio and albumin are effective risk factors on short and long-term mortality. However, when investigation of the roc analysis it was found that it has not significantly difference from CCI on long term mortality. Charlson Comorbidity Index does not include any of biochemical risk factors such as FT3/FT4 ratio and albumin. The new model, The Haseki Scoring Index (u-CCI) which combined with FT3/FT4 ratio and albumin, has better prognostic performance in short term mortality than the original CCI score. Albumin and low FT3/FT4 ratio may be assessed with the Charlson comorbidity index and this new model can be useful to predict short term mortality risk.

## 5. Conclusion

Low FT3/FT4 ratio and albumin could improve the prognostic performance of the original Charlson Comorbidity Index in short term mortality. The combined score may offer improvements in comorbidity summarization over existing scores. For this reason, index parameters can be changed in the future in predicting short and long-term mortality and biochemical tests such as albumin, FT3/FT4 ratio should be added.

## Supporting information

S1 Table The scoring of comorbidities, albumin and thyroid hormone index according to relative risk. (DOCX)
