# Supplementary material for: Serum albumin and FT3/FT4 ratio as additional co-morbidity parameters to predict mortality as a new approach: The Haseki Scoring Index (updated Charlson Comorbidity Index)
Source: PLoS One. 2022 Mar 14;17(3):e0264724. doi: 10.1371/journal.pone.0264724 (PMC8920220; doi:10.1371/journal.pone.0264724)
Supplement: S1 Table — (DOCX) [file pone.0264724.s001.docx]

**Supplement 1**: The scoring of comorbidities, albumin and thyroid hormone index according to relative risk

|  | 3 Month Mortality Score | 5 Year Mortality Score |
| --- | --- | --- |
| Age |  |  |
| Age (<50 / 50 - 59) | 1 | 3 |
| Age(<50 / 60 - 69) | 3 | 3 |
| Age(<50 / 70 -79) | 3 | 4 |
| Age(<50 / ≥80) | 4 | 5 |
| Diabetes Mellitus Without Endorgan Damage | 1 | 1 |
| Diabetes Mellitus With Endorgan Damage | 1 | 2 |
| Liver Damage | 1 | 1 |
| Solid Tumor Without Metastasis | 3 | 3 |
| Solid Tumor With Metastasis | 6 | 7 |
| AIDS | 15 | 22 |
| Chronic Renal Failure | 2 | 2 |
| Congestive Heart Failure | 1 | 2 |
| İschemic Heart Disease | 1 | 1 |
| Chronic Obstructive Pulmonary Disease | 1 | 2 |
| Peripheral Artery Disease | 1 | 3 |
| Cerebrovascular Disease | 1 | 1 |
| Dementia | 3 | 3 |
| Hemiplegia | 1 | 3 |
| Connective Tissue Disease | 1 | 1 |
| Leukemia | 5 | 5 |
| Lymphoma | 1 | 1 |
| Peptic Ulcer | 1 | 1 |
| Hypoalbuminemia | 4 | 3 |
| The Thyroid Hormone İndex (Ft3/Ft4 ≤2,27) | 3 | 2 |
